# Risk Attitude, Contract Arrangements and Enforcement in Food Safety Governance: A China’s Agri-Food Supply Chain Scenario

**DOI:** 10.3390/ijerph17082733

**Published:** 2020-04-15

**Authors:** Jing Hou, Linhai Wu, Bo Hou

**Affiliations:** 1 Business School, Jiangsu Normal University, Xuzhou 221116, China; houjing@jsnu.edu.cn; 2 Food Safety Research Base of Jiangsu Province, School of Business, Jiangnan University, Wuxi 214122, China; 3 School of Philosophy and Public Administration, Jiangsu Normal University, Xuzhou 221116, China; houbo@jsnu.edu.cn

**Keywords:** agri-food supply chain, food safety governance, risk, contract, economic field experiment

## Abstract

Frequent food safety problems in recent years have seriously affected China’s public health. The complexity, diversity, and technicality of food safety problems are intertwined, which constantly promotes the Chinese government and the food industry to explore effective food safety governance mechanisms. As the dominant form of vertical coordination in agri-food supply chain, contract farming is conducive to promoting farmers’ safe production behavior, improving food quality and safety, and ensuring public health. However, the low contract performance rate seriously restricts the effect of contract farming on reducing the risk of food safety in China. This paper empirically investigates the role of farmers’ risk attitude and contract arrangements in their enforcement. The data is derived from a household survey and economic field experiment of fruit farmers participating in contract farming in Anhui and Jiangsu provinces of China. We measure farmers’ risk attitude by using an economic field experiment, and examine how risk attitude and contract arrangements affect contract enforcement with a probit model. The results show that contract enforcement is significantly (1% level) influenced by farmers’ risk attitude. Farmers with greater risk aversion and loss aversion and farmers who are more accurate in evaluating probability information are more likely to fulfill the contract. Additionally, most contract arrangements have a significant effect on contract enforcement at the 5% level. Contract arrangements with floor pricing, bonus, and long duration are related to a higher rate of farmers’ contract performance. Furthermore, the factors influencing contract enforcement differ based on the levels of farmer’ risk attitude. The findings of the study may be conducive to formulating relevant agricultural policy to influence farmers’ decision-making and thus improving food safety and ensuring public health.

## 1. Introduction

Food safety governance is a common issue all over the world [1]. In recent years, the problem of food quality and safety in China is particularly severe [2]. China’s food safety incidents, such as Melamine milk powder, lean meat powder, Sudan red salted duck egg, and sick and dead pigs entering the market (the first three events involved the illegal addition of the harmful raw materials, the substances that can promote the growth of lean meat, and the carcinogenic chemicals. The event of dead pigs denotes that the dead pigs which should have been treated innocuously were illegally transported to the market for sale), have received particular public and media attention because they could cause severe health problems (more than 50 thousand people could get sick or die) [3]. Therefore, the issue of how to effectively reduce food safety risk is very important in front of the Chinese government [4]. As an international problem, the causes of food safety risk are complex and diverse, including natural and human factors [5]. The former is mainly subject to the changes of natural ecological environment and human scientific and technological forces, while the latter can be alleviated by improving the food safety governance mechanisms.

As one of the important governance mechanisms reducing the risk of food safety, contract farming has rapidly become the dominant form of vertical coordination in developed countries [6]. In recent decades, contract farming has become increasingly popular in China’s agri-food supply chain [7,8]. Contract farming is conducive to regulating farmers’ production behavior, promoting food safety, and ensuring public health [9,10]. For example, farmers participating in contract farming are required to use pesticides in accordance with the national regulations of pesticide use safety (such as the requirements for drug varieties, dosage, and withdrawal time), and agribusiness firms send technicians to guide and supervise farmers’ production behavior on a regular basis. Farmers who participate in contract farming can also access to new agricultural technologies [11]. It is thus clear that contract farming not only helps farmers to improve their production experience and awareness of safety production, but also restricts farmers’ irregular production behavior and promotes the quality and safety of agricultural products. However, the enforcement of the contract is not optimistic in China’s agri-food supply chain [12,13]. For example, Guo [13] found that the farmers’ contract performance rate was only 38 percent in a survey made in China. The issue of contract enforcement not only affects the sustainable expansion of contract farming in China, but also affects the role of contract farming in reducing the risk of food safety. Therefore, how to ensure the enforcement of the contract in China’s agri-food supply chain is of great significance to improve the quality and safety of agricultural products.

In China, contract arrangements between farmers and processing or distribution firms are the main types of vertical coordination [14]. Farmers prefer and perform differently based on different contract farming models (i.e., they have autonomous choice in enforcement) [15]. The existing literature has studied impact factors of contract enforcement, which include reputation mechanism [16], specific investments [17,18], contract terms [14,19], and transaction costs [20,21]. However, the literature mainly focuses on the impact of external factors on contract performance and generally presupposes that the behavioral agents are rational, ignoring the agents’ personality traits such as risk attitude [22,23]. In reality, decision-making is a process of complex cognitive operations influenced by personal as well as environmental variables [24]. Researchers suggest that risk attitude plays an important role in farmers’ investment decisions and agricultural production decisions [25,26]. Beyond decisions, risk perceptions may determine farmers’ health and coping behaviors, both toward the business and the environment [27,28,29]. Thus, it is more appropriate to incorporate farmers’ risk attitude and contract arrangements into the study of contract enforcement. In determining the relationship between risk attitude and agricultural decisions, most empirical researches in the literature typically use two methods when estimating individual risk attitude [30]. One is to depend on the assumption of objective function and advanced econometric technique to impute the coefficient of risk aversion that will fit the model; however, the assumption of a utility function form and arbitrary heuristics could cause bias. The other method is using wealth as a proxy for risk aversion; but this method could be problematic as it could potentially undermine the role of risk attitude in farmers’ decisions. One contribution to the existing literature is that all risk attitude parameters used in the analysis of contract enforcement are being elicited from an economic field experiment.

This paper attempts to examine the effect of farmers’ risk attitude and contract arrangements on their enforcement in the scenario of China’s agri-food supply chain, with the goal of exploring the governance path for effectively preventing food safety risk. Our data is from a household survey and economic field experiment conducted in Anhui and Jiangsu provinces of China. In this study, we first measure farmers’ risk attitude from an economic field experiment, and then extend their experiment results to the contract performance decisions. The enforcement of the contract in agri-food supply chain is a relatively less researched dimension of contract farming and food safety, and we expect the paper to be a good addition to the existing literature.

## 2. Conceptual Framework

The contract enforcement decision for a farmer is a tradeoff between the revenues and the costs [31]. The farmer’s default revenues (*R*) are determined by the default quantity (*Q*) times the wedge between the contracted price (*p_c_*) and the actual market price (*p_m_*); thus, we can express it as: $R=Q*(p_{m}-p_{c})$. We assume the contractor will not trade with a farmer over some period of time if farmers’ default behavior has been exposed. The farmer’s default costs (*C*) depend on the value of losses (*l*) that result from termination or non-renewal of the contract, the damage of the farmer’s reputation in local business, and the probability of exposure and punishment for defaulting contract (*P*). Thus, we can express these default costs as: $C=f(P)*\sum_{i}^{n}c_{i}/{\left(1+\delta \right)}^{i}$, where $c_{i}$ is the value of farmer’s losses in the period *i*, *δ* is the discount factor, and *P* is the probability of exposure and punishment that is affected by the efficiency of legal system and supervision and farmer’s risk attitude. The probability that the farmer is forced to pay compensation for losses resulting from contract default is determined by the cost of contract enforcement for the agribusiness firm and the adequacy of the legal system. The costs of contract enforcement are time, effort, and money that must be spent to take legal action; these are affected by efficiency of the legal system, contract provisions and design, and characteristics of the firm or farmer [14]. Following the above discussion, we can specify the farmer’s contract enforcement strategy.

When the market price exceeds the contracted price, the farmer will weigh the short-term benefits achieved by defaulting contract (i.e., the default revenues) and the future utility stream of losses (i.e., the default costs). A farmer has incentive to default the contract only when the default revenues exceed the default costs. Considering that the default quantity is relatively small in reality, the decision-making in contract default or performance mainly depends on the default costs; the higher the costs, the greater the probability of contract performance.

From the above discussion, we can infer that the value of *l*, *P*, and the wedge between $p_{m}$ and $p_{c}$ will affect farmers’ contract performance rate. Given the state of the legal system and market conditions, these values are determined by contract arrangements, such as contract form, contract type, or contracted price [14]. Notably, farmers’ risk attitude may affect the subjective utility of costs resulting from contract default and thus influence the contract performance decisions. For example, if a farmer is risk loving or fails to accurately evaluate probability information, he or she tends to undervalue the probability that the contractor will discover the default behavior. Consequently, the potential default costs decrease, leading to a substantial increase in the likelihood of contract default.

To analyze the effect of farmers’ risk attitude and contract arrangements on the enforcement of the contract in China’s agri-food supply chain, we developed a straightforward reduced form model as follows:

(1) $$CE_{i}=F(risk_{i},arrangements_{i},Z_{i};\eta )+\varepsilon_{i}$$

where *CE* refers to contract enforcement and is measured by farmers’ contract performance decisions; *risk* and *arrangements* denote key explanatory variables and *Z* represents a vector of control variables that are factors influencing contract enforcement. The variables are defined below.

The dependent variable, *CE*, is indicated by a discrete value of 0 or 1 and is derived from contracted farmers’ response to the survey questions. The value is 1 if farmer *i* fulfills the contract, and 0 otherwise. When the market price exceeds the contracted price, the farmer may sell a portion of products to the market. Moreover, we also observe whether the farmer carries out standardized production according to the contractual stipulation.

The independent variable, *risk*, is defined as farmers’ risk attitude. The common approach uses expected utility (EU) to measure individual risk attitude, in which risk aversion is the sole parameter that determine the shape of the utility function; however, the existing literature suggests that farmers’ risk attitude is better captured by prospect theory rather than expected utility theory in the context of agricultural decision-making [25,30]. As Liu [25] points out, Chinese farmers generally have an expected income level they want to achieve, and they are more sensitive to losses than to gains at the expected income level; thus, parameters other than risk aversion, such as loss aversion, should also be considered in the research. In prospect theory, three variables (i.e., risk aversion, probability weighting, and loss aversion) jointly determine the shape of the utility function [32]. A farmer’s utility under prospect theory is defined below:

(2) $$PT(x,p;y,1-p)=\left\{ \begin{array}{cccc} v(y)+\pi (p)(v(x)-v(y)), & x>y>0 & or & x<y<0\\ \pi (p)v(x)+\pi (1-p)v(y), & x<0<y & & \end{array}\right.$$

In equation (2), PT(x,p;y,1−p) denotes the expected value over binary monetary outcome *x* and *y* with corresponding probabilities *p* and 1 − *p*, respectively. Furthermore, a two-part power function assigns a value for gains (*x* > 0) and losses (*x* < 0) separately:

(3) $$v(x)=\{\begin{array}{cc} x^{\sigma } & ,x\geq 0\\ -\lambda {\left(-x\right)}^{\sigma } & ,x<0 \end{array}$$

where *σ* reflects the curvature of the value function and can be interpreted as a proxy for risk aversion (The higher the value function curvature (*σ*), the lower the levels of risk aversion. The individual is risk-averse if *σ* < 1, risk-loving if *σ* > 1, and risk-neutral if *σ* = 1.); *λ* reflects the degree of loss aversion. Additionally, we assume that farmers place a decision weight on probability information *p* that reflects the desirability of uncertain events. Thus, the probability weighting function is defined below:

(4) $$\pi (p)=\exp\left[-{\left(-\ln p\right)}^{\alpha }\right]$$

where *α* represents a proxy for probability weighting [33]. (*α* reflects the accuracy of assessing probability events, and is associated with overweighting small probability events and underweighting large probability events. *α* < 1 implies that individual does not correctly estimate probabilities, resulting in an inverted s-shaped probability weighting function.) The smaller *α* is, the larger the departure from linear probability weighting and hence, the stronger the tendency to overweight small probabilities and underweight large probabilities. Specially, if α=1 and λ=1, the above specification reduces to the standard expected utility specification.

Defaulting contracts is risky behavior for farmers because farmers may suffer a large loss resulting from default; thus, we assume that a more risk-averse farmer has a higher tendency to fulfill the contract. The loss aversion parameter is assumed to be positively related to contract enforcement. Farmers with higher levels of loss aversion tend to attach more value to the penalties and losses resulting from contract default, and thus have a higher tendency to fulfill the contract. Additionally, we expect that probability weighting parameter is positively related to contract enforcement. Generally, farmers who are unable to correctly appraise probability information are prone to underestimate the probability of exposure and punishment by contractor and thus have a low likelihood of contract performance.

The independent variable, *arrangements*, is defined as contract arrangements or terms, which is an important factor influencing farmers’ decision-making [14,18]. In this paper, we include four indicators that describe the contract arrangements: (a) the form of contract (*form*), (b) contract pricing mechanism (*pricing*), (c) contract duration (*duration*), and (d) bonus clause (*bonus*). The variable *form* measures the form of contract established between the contractor and the farmer; it takes on the value of 1 if the contract form is oral, and it is 0 if the contract form is written contracts. If a farmer signs an oral contract with an agribusiness firm, the court is difficult to determine the responsibility of default, which may lead to a decrease in farmers’ default costs [14]; thus, we assume that the probability of contract performance is higher with written contracts than with oral contracts. The variable *pricing* refers to the contract pricing mechanism specified in the contract; it has a value of 1 if the mechanism is floor pricing (contract with a floor pricing mechanism offers farmers a minimum price at the beginning of planting; at the time of the delivery, the contractors will use the minimum price if the market price is lower than the minimum price, otherwise they will use the market price), and it is 0 if the mechanism is fixed pricing. We expect that the floor pricing mechanism is associated with a high rate of contract performance because it provides farmers with income guarantees [14]. The variable *duration* denotes the length of the contract between the contractor and farmers. Farmers who sign long-term contracts with the contractor tend to consider the adverse effects of contract default from their own long-term interests; the longer the duration of the contract, the greater the losses from defaulting contract. Thus, we assume that contract duration is positively related to the probability of contract performance. The variable *bonus* is binary and has a value of 1 if the contractor offered a bonus to farmers who complied with the contract and 0 otherwise. We expect that contract terms with a bonus are associated with a high rate of contract performance, because such bonus clause will increase the farmers’ income and improve the enthusiasm of farmers cooperating with contractor [18].

In addition to risk attitude and contract arrangements, contract enforcement may be influenced by other factors such as demographic and socio-economic characteristics. The rest of the control variables consist of the age of household head (*age*), the education of household head (*education*), household labor (*labor*), and family asset (*asset*), farm scale (*scale*), planting experience (*experience*), the distance from farm to contractor (*distance*), the fluctuation of market price (*fluctuation*), and regional differences (*region*).

## 3. Materials and Methods

### 3.1. Data Collection

The data was derived from a household survey and economic field experiment of Chinese fruit farmers conducted in 2017. We chose apple and pear, two popular and common fruit in China as representative commodities. China produces more than 20 million tons of apples per year, making it the largest producer in the world. China’s pear production reached 18.7 million tons in 2016, accounting for 75% of world pear production. Therefore, the study on Chinese fruit industry has an important meaning.

Our study was in Anhui province and Jiangsu province. The study county in each province was selected based on a random pre-selection, which was followed by the purposive selection of counties with high rate of contract participation. Specifically, in our survey we chose two counties, Dangshan and Fengxian, which are located in Anhui Province and Jiangsu Province, respectively. In each county that was selected, we randomly picked two towns. In every town, we randomly picked two villages, and in each village we sampled 30 fruit farmers participating in contract farming. Notably, a pilot survey was conducted before formal interview. Prior to the interview process, we conducted several telephone calls with an agribusiness firm to explain the design and the purpose of the farmer survey, its implications, the interview approach, and the sample selection procedures; then we randomly interviewed ten target population—fruit households who have contracted with the agribusiness firm. After the pre-survey was done, the effectiveness of the queries and statements in the questionnaire is tested and improved.

The survey included demographic and socioeconomic information such as age, education, risk attitude, household labor, family asset, farm scale, production and sales details, and contract arrangements. In order to ensure smooth interaction between interviewers and farmers, easy-to-answer questions were presented first in the survey, such as farmers’ individual and household characteristics. The second part of the survey covered the main topic for this study and involved questions and statements designed to assess contract enforcement information. The last part of the survey included a field experiment addressing risk attitude. We applied for the ethical approval of the research involving human participants. The questionnaire was based on a literature review, related theories, peer review and revision, in combination with results of pre-survey, which indicates that it has a good content validity. To test the reliability of the questionnaire, we used Cronbach’s alpha as an indicator to test the reliability of samples. We observed that the overall Cronbach’s alpha coefficient is 0.71, implying that the questionnaire has a good and stable reliability.

In total, 235 usage observations were obtained, of which 118 (50.2%) farmers growing apples and 117 (49.8%) farmers growing pears.

### 3.2. Experimental Measure of Risk Attitude

Following Liu [25] and Tanaka et al. [34], we elicited farmers’ risk attitude by an economic field experiment. The field experiment on risk attitude took the form of a “switching Multiple Price List” (sMPL) design [35,36,37]. MPL is the standard format in which the participant observes a fixed array of paired options and chooses one for each row. sMPL varies the standard MPL by asking the participant to simply choose which row he or she wants to switch at, assuming monotonicity of the underlying preferences to fill out the remaining choices for the participant [35].

The risk attitude experiment was designed to estimate three prospect theory parameters. The experiment is illustrated in Table 1. There are three series of paired lotteries. Each paired lottery consists of a safe reward (Option A) and a risky reward (Option B). Take Row 1 for example: If farmers choose option A, they have a 30% probability of winning 20 Yuan and a 70% probability of winning 5 Yuan; if farmers choose option B, there is a 10% probability of winning 34 Yuan and a 90% probability of winning 2.5 Yuan. Farmers must made a choice between two options in each series.

Across the risk attitude experiment, farmers aggregately completed 35 decision tasks. At the end of the experiment, one pair of lotteries was randomly chosen to be paid with real monetary reward. The real monetary reward was expected to encourage participants to reveal their true preferences [35]. (Notably, the experimental tasks took an average of 10 minutes and the average reward was 35 Yuan.) Specifically, we prepared two pairs of numbered cards. After making all 35 choices in the experiment, participants were asked to first draw one card out of the first pair that contains 35 numbered cards. The number on that card determines which row would be paid the real money. They then drew another card out of the second pair that contains 10 cards numbered 1 through 10. Depending on the lottery they had chosen for that particular row, their rewards were determined by the second numbered card.

We estimated risk attitude parameters based on the farmers’ choices made in the experiment. Specifically, experiment results from Series 1 and Series 2 were used to jointly estimate the curvature of the utility function in the positive domain (*σ*) and the probability weighting parameter (*α*); experiment results from Series 3 were used to estimate the loss aversion parameter (*λ*). For any participant who switch at row N, we could determine whether he or she prefers Option B over Option A at Row N and prefers Option A over Option B at row N-1. Thus, a set of two inequalities from this switching point could be obtained. For instance, if a participant switched from Option A to Option B at row 7 for both Series 1 and Series 2, then the following inequalities should be satisfied:

(5) $$\begin{array}{l} 5^{\sigma }+\exp[-{\left(-\ln0.3\right)}^{\alpha }](20^{\sigma }-5^{\sigma })>2.5^{\sigma }+\exp[-{\left(-\ln0.1\right)}^{\alpha }](62.5^{\sigma }-2.5^{\sigma })\\ 5^{\sigma }+\exp[-{\left(-\ln0.3\right)}^{\alpha }](20^{\sigma }-5^{\sigma })<2.5^{\sigma }+\exp[-{\left(-\ln0.1\right)}^{\alpha }](75^{\sigma }-2.5^{\sigma })\\ 15^{\sigma }+\exp[-{\left(-\ln0.9\right)}^{\alpha }](20^{\sigma }-15^{\sigma })>2.5^{\sigma }+\exp[-{\left(-\ln0.7\right)}^{\alpha }](32.5^{\sigma }-2.5^{\sigma })\\ 15^{\sigma }+\exp[-{\left(-\ln0.9\right)}^{\alpha }](20^{\sigma }-15^{\sigma })<2.5^{\sigma }+\exp[-{\left(-\ln0.7\right)}^{\alpha }](34^{\sigma }-2.5^{\sigma }) \end{array}$$

We could calculate the intervals of *σ* and *α* that satisfy this pair of inequalities by using the combination of switching points from Series 1 and Series 2; then, we followed Liu [25] and Tanaka et al. [34] to take the midpoint of each interval as the point estimate. After completing the estimates of *σ* and *α*, the inequalities involving *λ* could be written out by using the switching point from Series 3. For instance, if a participant switched from Option A to Option B at row 7 for Series 3, then the following inequalities should be satisfied:

(6) $$\begin{array}{l} \exp[-{\left(-\ln0.5\right)}^{\alpha }](-4^{\alpha }\lambda +0.5^{\alpha })>\exp[-{\left(-\ln0.5\right)}^{\alpha }](-7^{\alpha }\lambda +15^{\alpha })\\ \exp[-{\left(-\ln0.5\right)}^{\alpha }](-4^{\alpha }\lambda +0.5^{\alpha })<\exp[-{\left(-\ln0.5\right)}^{\alpha }](-5.5^{\alpha }\lambda +15^{\alpha }) \end{array}.$$

Thus, we could obtain the range of *λ* by solving for the above inequalities. Similarly, we used the midpoint of the interval as the point estimate of *λ*.

### 3.3. Empirical Methods

We used a probit model with a maximum likelihood estimator to explore the effect of farmers’ risk attitude and contract arrangements on contract enforcement. The dependent variable, *CE*, refers to contract enforcement. The independent variables include the key explanatory variables associated with farmer *i*’s risk attitude and contract arrangements, and a vector of control variables related to farmer *i*’s demographic and socio-economic characteristics.

The estimating equation is expressed below:

(7) $$\mathrm{Pr}(CE_{i}=1)=F(\eta^{\prime }x_{i})=\int_{-\infty }^{\eta^{\prime }x_{i}}\frac{1}{\sqrt{2\pi }}\exp(-\frac{z^{2}}{2})dz$$

where *F*( ) follows a cumulative normal distribution function giving the probability of contract performance and *x_i_* is the hypothesized explanatory variables. The individual likelihood for farmer *i* is described as follows:

(8) $$p(CE_{i})=F{\left(\eta^{\prime }x_{i}\right)}^{CE_{i}}{\left[1-F(\eta^{\prime }x_{i})\right]}^{\left(1-CE_{i}\right)}$$

Thus, we were able to obtain the likelihood function for all observations by assuming independence across individuals:

(9) $$L=\prod_{i=1}^{n}p(CE_{i})=\prod_{i=1}^{n}F{\left(\eta^{\prime }x_{i}\right)}^{CE_{i}}{\left[1-F(\eta^{\prime }x_{i})\right]}^{\left(1-CE_{i}\right)}$$

The probit model was estimated by Stata 15.0 software (StataCorp LP, TX, USA).

## 4. Results and Discussion

### 4.1. Data Analysis

**Figure 1:**
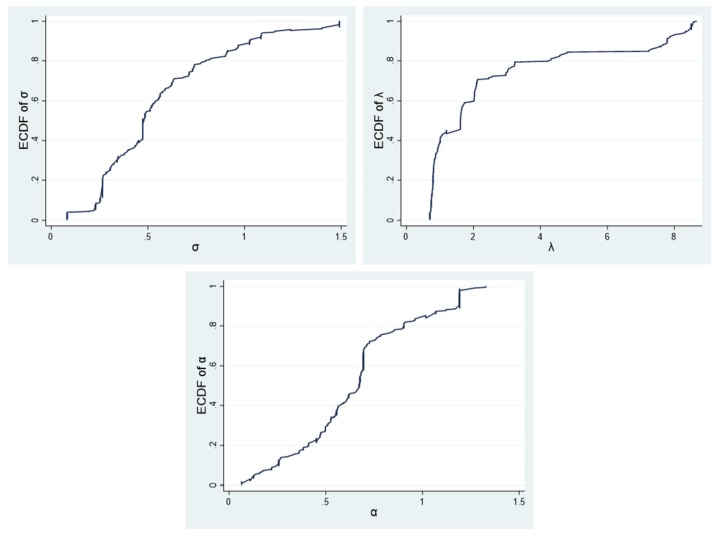
Cumulative distribution function plots of risk attitude’ parameters.

**Figure 2:**
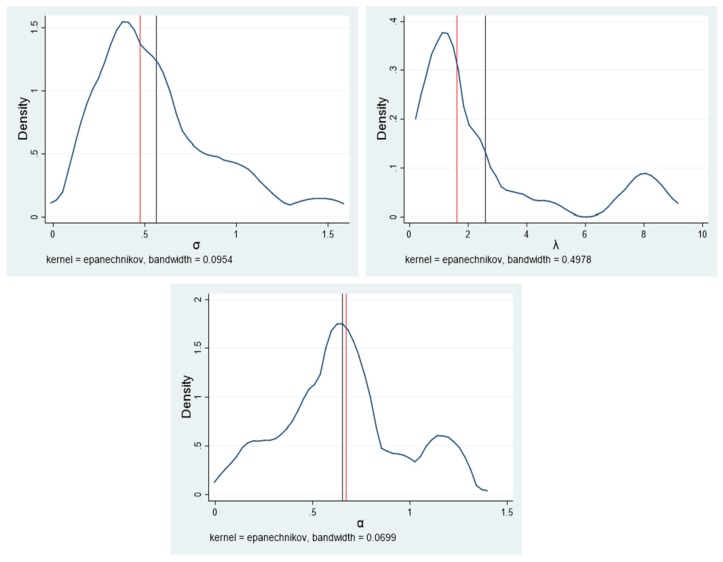
Kernel density estimation plots of risk attitude’ parameters. Notes: In the figure, the black line represents the mean value, and the red line represents the 50th quantile (i.e., the median).

We carefully estimated each individual’s risk attitude parameters. The cumulative distribution function plots and kernel density estimation plots of the risk attitude’ parameters are shown in Figure 1 and Figure 2, respectively. The estimate of the risk aversion parameter *σ* (Mean = 0.56; SD = 0.33) was less than 1, suggesting that average Chinese fruit farmer is risk-averse in the gain domain. Notably, the estimate of *σ* was higher than that obtained in similar study in Africa (e.g., [37]). This comparison implies that the average Chinese fruit farmer in our sample is less risk-averse in the gain domain than the average cattle farmer in West Africa. Furthermore, the estimates of the loss aversion parameter *λ* (Mean = 2.59; SD =2.54) was more than 1, implying that the average Chinese fruit farmer appears to pay more attention to losses than gains. Additionally, the estimate of the probability weighting parameter *α* (Mean = 0.66; SD =0.30) was less than 1, which suggests that the average Chinese fruit farmer is unable to correctly assess probability information.

**Figure 3:**
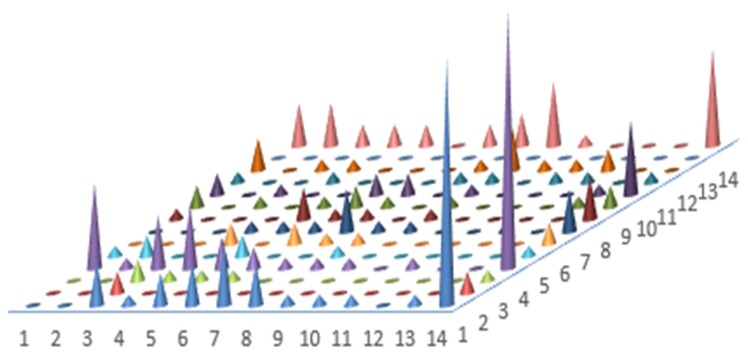
Distribution of switching point in series 1 and 2.

Figure 3 shows the distributions of choices made by farmers in Series 1 and 2. The numbers in the axes correspond to the switching points in Series 1 and 2. The height of a cone denotes the number of farmers who switched at that particular combination of switching points in Series 1 and 2. We observe that farmers in this study have a wide range of risk attitude.

Table 2 provides the description and summary statistics of variables. As shown in Table 2, the rate of contract performance in our survey was about 64% (SD = 0.46). The average household head was 49 (SD = 9.28) years old and had seven years of formal schooling. The average years of planting fruit for contracted farmers were about 15 (SD = 8.84) years. With respect to the contract terms, only about 10% (SD = 0.30) of the respondents used oral contracts; approximately 69% (SD = 0.46) of the respondents used a float pricing mechanism; the average length of contract was about 2.3 (SD = 1.17) years; and about 78% (SD = 0.42) reported that contractor offered a bonus clause. Additionally, an average distance from farm to contractor of 21 (SD = 11.61) km was observed.

### 4.2. Regression Results

The probit estimates are reported in Table 3. In order to ascertain the magnitude of the effects of the independent variables on contract enforcement, we calculated and presented the marginal effects. We focus first on the coefficient estimates of the risk attitude parameters. Consistent with our expectations, the risk aversion parameter (*σ*) had a significant negative effect on contract enforcement at the 1% level. A smaller *σ* is associated with greater risk aversion. The result indicates that farmers with higher levels of risk aversion were more likely to fulfill the contract, as every unit decrease in *σ* was associated with a 67% increase in the likelihood of contract performance. In view of the risk uncertainties in the market, particularly in the market price and market channel, it is risky for farmers to default a contract; thus, a risk-averse farmer tended to fulfill the contract in the sales phase. Also in accordance with our expectations, the loss aversion parameter (*λ*) showed a significant (1% level) positive effect on contract enforcement. For every additional unit of *λ*, farmers were almost 6% more likely to fulfill the contract. If a farmer had lower levels of loss aversion, he or she would pay less attention to contract default costs such as penalties; this may cause farmer to undervalue the consequences, leading to a high probability of defaulting contract. We also found a significantly (1% level) positive correlation among probability weighting parameter (*α*) and contract enforcement: every additional unit of *α* would increase the probability of contract performance by 33%. This result supports the theoretical hypothesis regarding probability weighting. Based on the results of several studies, individuals tended to underweight likely but undesirable events and to overweight unlikely but desirable events [34,36,37]. Given that contracting firms regularly send technicians to inspect and sample products, the probability of being discovered by the firm was relatively high. Driven by such psychological characteristic, farmers who fail to accurately evaluate probability information tended to underestimate the probability that the contractor would discover their default behavior and thus had a higher motivation of contract default. The above results imply that farmers’ risk attitude plays an important role in their contract performance decisions.

With respect to the contract arrangements, we observed that the variable *pricing* was significantly (5% level) and positively related to contract enforcement. Farmers who signed a contract with floor pricing were almost 10% more likely to fulfill the contract than farmers who signed a contract with fixed pricing. The variable *duration* was observed to have a significant (5% level) positive effect on contract enforcement. The result implies that the longer the duration of the contract, the more likely the farmers were to fulfill the contract, as every additional year of contract duration was associated with a 3% increase in the probability of contract performance. Additionally, the variable *bonus* had a significant (1% level) positive effect on contract enforcement. Farmers who signed a contract with bonus clause were 15% more likely to fulfill the contract than farmers who signed a contract without bonus clause. These results are consistent with our expectations and the findings of other studies [14,18], implying farmers’ contract performance decisions are affected by contract arrangements of agri-food supply chain.

However, contrary to our expectations, the variable *form* showed no significant positive effect on contract enforcement. In China, the enforcement of oral contracts may rely on the farmers’ network and norms as the majority of cooperatives are organized by the farmers themselves. Many middlemen live in rural areas and belong to the same rural social networks as farmers, so they are able to assess the farmers’ reliability [14,38]. From this point of view, an oral contract does not necessarily decrease the rate of contract fulfillment.

The other statistically significant results in Table 3 are also consistent with our expectations. For example, the variable *age* had a significant positive effect on contract enforcement. Every additional year of age would imply a 0.6% increase in the probability of contract performance. The variable *experience* showed a significant negative effect on contract enforcement: Every additional year of planting fruit was associated with a 0.4% increase in the probability of contract performance. Additionally, the variable region was observed to have a significant negative effect on contract enforcement.

It is worth noting that the variable distance, which reflects to transaction costs, showed no significant effect on contract enforcement. This result is contrary to our expectations. Although the literature suggests that transaction costs should be related to contract enforcement [20,21], our empirical findings do not reveal that relationship. This result may be because the sample is not homogenous. In next subsection, we further explored the group differences in the factors influencing the contract enforcement.

### 4.3. Further Discussion

We expect different groups of farmers to use different criteria when making contract performance decisions. Following Pennings and Leuthold [39], we adopted a cluster analysis by using squared Euclidean distances and Ward’s method to test for heterogeneity. The risk attitude parameters (σ, λ, and α) were included in the cluster analysis. We observed two distinct subsamples. Subsample 1 comprised 141 farmers with a relatively high degree of risk propensity. Subsample 2 comprised 94 farmers with a relatively low degree of risk propensity. To explore the differences between these two subsamples, we analyzed the characteristics of the farmers regarding the levels of risk-propensity by using the Mann–Whitney U test [40]. The two subsamples significantly differed regarding farmers’ contract performance decisions (Prob = 0.000). Therefore, we divided the sample into two segments and estimated the parameters for each segment with a probit model. The regression results are presented in Table 4.

From Table 4, we observed that the variable *pricing* showed a significant effect on contract enforcement in both subsamples. Notably, we found some differences in the two subsamples. For example, the contract arrangement variables *form*, *duration*, and *bonus* had a significant effect on contract enforcement for less risk-propensity farmers, but were not significant for more risk-propensity farmers. We also observed that the variable *distance* was not significant for more risk-propensity farmers, but had a significant effect on less risk-propensity farmers. The results imply that for farmers who have low degree of risk propensity, contract arrangements (e.g., contract form, the duration of contract, and bonus) and transaction costs (e.g., the distance from farm to contractor) do play a role in farmers’ contract performance decisions. This result is consistent with the findings of Guo and Jolly [14], Kumar et al. [18], and Haji [20]. With respect to farmers who have high degree of risk propensity, however, the mechanisms of contract arrangements and transaction costs do not play a significant role. One possible reason is that farmers with greater risk propensity prefer to take risks and pay less attention to the consequences, leading to the role of traditional mechanisms such as reputation in contract enforcement for farmers having less influence on behavior. The results imply that reducing transaction costs may lead to an increase in contract performance, but farmers’ risk propensity weaken their role in the enforcement of the contract.

### 4.4. Robustness Checks

#### 4.4.1. Robustness Check by Excluding the Outlier of Preferences

In this survey, about 7.2% of farmers chose either only Option A or only Option B during the experiment. In the risk attitude experiment, there were cases of “never switch” or “switching at row 1” for all of the three series. One concern that arises is that these farmers might not treat the experiment seriously, or had been innumerate and thus not understand how the experiment works [25]. If this is the case, they added noise to the estimates. As a robustness check, we excluded those individuals from the full sample.

The regression results are reported in Table 5. We found that the interpretations of the coefficients did not vary too much from regression results with the full sample. Particularly, the estimates of risk attitude parameters (*σ*, *λ*, and *α*) and contract arrangements parameters (*pricing*, *duration*, and *bonus*) remained robust to exclude the outlier. The results suggest a strong role of risk attitude and contract arrangements in farmers’ contract performance decisions.

#### 4.4.2. Robustness Check by Using the Survey Questions of Preferences

In this study we measured farmers’ risk attitude by using field experiment. A concern regarding this study might be that we imposed too strong a functional form assumption on the value function when deriving the risk attitude. Therefore, we conducted a robustness check by using the survey questions of risk attitude.

During our survey, in addition to eliciting farmers’ risk attitude by experiment, we also asked farmers to rank their risk attitude from most risk averse to most risk loving on a 1–5 Likert scale. The smaller the value, the higher the degree of risk aversion. We found that, on average, the level of risk aversion was 2.221 (SD = 1.122). This result may suggest that the average fruit farmer in our sample is risk averse.

Table 6 presents the regression results of survey questions. We observed that the variable *risk attitude* had a significant negative effect on contract enforcement. A smaller *risk attitude* is associated with greater risk aversion. The result indicates that the probability of farmers’ contract performance increase as the levels of risk aversion increase, which is consistent with our main findings. Additionally, the results also showed a significant relationship between contract arrangements (the variables *pricing*, *duration*, and *bonus*) and contract enforcement.

## 5. Conclusions

This paper studied how farmers’ risk attitude and contract arrangements affected their enforcement in agri-food supply chain. A better understanding of farmers’ risk attitude and contract arrangements is of great significance for designing more effective agricultural policy regarding contract enforcement, thus better promoting food safety and ensuring public health. To achieve our objective, we conducted a survey and economic field experiment on Chinese fruit farmers and focused on estimating a probit model of contract enforcement. The most important result is that contract enforcement is significantly affected by farmers’ risk attitude and contract arrangements. Specifically, farmers with higher levels of risk aversion and loss aversion are more willing to fulfill contracts; however, farmers who are less accurate in evaluating probability information are less likely to fulfill a contract. Contract terms with floor pricing, bonus, and long duration are related to a high-performance level. Additionally, our results show that the groups of farmers have different decision structures, and the factors influencing contract enforcement differ based on the levels of farmer’ risk attitude. Particularly, the role of contract arrangements and transaction costs in contract enforcement may be weakened for more risk-propensity farmers but do play a role for less risk propensity farmers.

In China, private mechanism design is an alternative to legal mechanisms. The combination of litigation costs, poor third party verifiability, and ineffective contract law make formal means of enforcement costly or ineffective [14]. The implication is that policy-making on improving the stability of contract should pay attention to the role of private contract enforcement mechanisms. Contract arrangements such as floor pricing and bonus provided by the contractor are conducive to increasing farmers’ contract performance rate and thus to promoting food safety. Furthermore, the policy-making on contract enforcement should pay attention to the influence of farmers’ risk attitude. Providing farmers more income security and policy insurance regarding fruit planting may mitigate their risk propensity and increase contract performance rate. Additionally, it is necessary to disclose food safety risk to farmers and actively publicize the role of contract farming in ensuring food safety, so as to improve farmers’ awareness of food safety and restrict their irregular production behavior.

Notably, our study may be limited as we only study the influencing factors of contract enforcement by farmers, but not by the contractor. Future research on contract enforcement should consider the factors influencing agribusiness firms’ contract performance decisions.

## Figures and Tables

**Table 1:** Design of risk attitude experiment.

		Option A		Option B	
**Series**	**Row**	**30% Probability**	**70% Probability**	**10% Probability**	**90% Probability**
1	1	20￥	5￥	34￥	2.5￥
	2	20￥	5￥	37.5￥	2.5￥
	3	20￥	5￥	41.5￥	2.5￥
	4	20￥	5￥	46.5￥	2.5￥
	5	20￥	5￥	53￥	2.5￥
	6	20￥	5￥	62.5￥	2.5￥
	7	20￥	5￥	75￥	2.5￥
	8	20￥	5￥	92.5￥	2.5￥
	9	20￥	5￥	110￥	2.5￥
	10	20￥	5￥	150￥	2.5￥
	11	20￥	5￥	200￥	2.5￥
	12	20￥	5￥	300￥	2.5￥
	13	20￥	5￥	500￥	2.5￥
	14	20￥	5￥	850￥	2.5￥
Series	Row	90% probability	10% probability	70% probability	30% probability
2	1	20￥	15￥	27￥	2.5￥
	2	20￥	15￥	28￥	2.5￥
	3	20￥	15￥	29￥	2.5￥
	4	20￥	15￥	30￥	2.5￥
	5	20￥	15￥	31￥	2.5￥
	6	20￥	15￥	32.5￥	2.5￥
	7	20￥	15￥	34￥	2.5￥
	8	20￥	15￥	36￥	2.5￥
	9	20￥	15￥	38.5￥	2.5￥
	10	20￥	15￥	41.5￥	2.5￥
	11	20￥	15￥	45￥	2.5￥
	12	20￥	15￥	50￥	2.5￥
	13	20￥	15￥	55￥	2.5￥
	14	20￥	15￥	65￥	2.5￥
Series	Row	50% probability	50% probability	50% probability	50% probability
3	1	12.5￥	−2￥	15￥	−10￥
	2	2￥	−2￥	15￥	−10￥
	3	0.5￥	−2￥	15￥	−10￥
	4	0.5￥	−2￥	15￥	−8￥
	5	0.5￥	−4￥	15￥	−8￥
	6	0.5￥	−4￥	15￥	−7￥
	7	0.5￥	−4￥	15￥	−5.5￥

**Table 2:** Description and summary statistics of variables.

Variable	Variable Definitions	Mean	SD
Contract enforcement	If farmers perform the contract =1, else = 0	0.636	0.482
Risk aversion (*σ*)	The curvature of the value function	0.564	0.326
Loss aversion (*λ*)	The degree of loss aversion	2.585	2.542
Probability weighting (*α*)	The accuracy of assessing probability events	0.655	0.303
Form	If contract form is oral = 1, else = 0	0.102	0.303
Pricing	If contract offers a floor price = 1, else = 0	0.689	0.464
Duration	The length of contract between the contractor and farmers (years)	2.332	1.173
Bonus	If firm offers a bonus to farmers who complies with the contract = 1, else = 0	0.779	0.416
Age	Age of household head in years	49.098	9.279
Education	Years of formal education of household head	7.119	2.658
Labor	Number of household labor force	2.953	1.014
Asset	Area of house or apartment (10^2^ square meters)	2.248	1.953
Scale	Area of planting (mu)	5.177	3.871
Experience	Years of planting fruit	14.598	8.841
Fluctuation	The regional market prices variance over the past five years	0.323	0.044
Distance	Distance from farm to contractor (km)	20.983	11.610
Region	1 denotes Jiangsu province, and 0 denotes Anhui province	0.502	0.501

**Table 3:** Estimation results of probit model.

Variable	Marginal Effect	(Std. Err.)
Risk aversion (*σ*)	−0.674 ***	(0.035)
Loss aversion (*λ*)	0.058 ***	(0.012)
Probability weighting (*α*)	0.333 ***	(0.053)
Form	0.086	(0.064)
Pricing	0.105 **	(0.045)
Duration	0.029 **	(0.015)
Bonus	0.154 ***	(0.042)
Age	0.006 **	(0.002)
Education	0.005	(0.007)
Labor	−0.003	(0.015)
Asset	0.005	(0.007)
Scale	−0.001	(0.004)
Experience	−0.004 *	(0.002)
Fluctuation	0.087	(0.480)
Distance	−0.001	(0.001)
Region	−0.089 **	(0.037)
Observations	235	
Pseudo-Log likelihood	−49.376	

Notes: Single, double, and triple asterisks (*, **, and ***) denote significance level of 0.10, 0.05, and 0.01, respectively.

**Table 4:** Subsample Regressions Based on Risk Attitude.

Variable	Farmers with High Degree of Risk Propensity		Farmers with Low Degree of Risk Propensity	
	Marginal Effect	(Std. Err.)	Marginal Effect	(Std. Err.)
Form	0.041	(0.120)	0.333 ***	(0.126)
Pricing	0.180 *	(0.094)	0.181 **	(0.091)
Duration	0.019	(0.035)	0.058 *	(0.032)
Bonus	0.082	(0.097)	0.120 *	(0.072)
Age	0.005	(0.006)	−0.001	(0.006)
Education	−0.012	(0.014)	0.005	(0.017)
Labor	0.025	(0.040)	0.003	(0.049)
Asset	−0.025	(0.026)	0.002	(0.018)
Scale	−0.001	(0.012)	0.006	(0.008)
Experience	−0.009 *	(0.006)	−0.003	(0.005)
Fluctuation	−0.490	(1.006)	−0.562	(1.129)
Distance	−0.006	(0.004)	−0.009 ***	(0.003)
Region	−0.089	(0.083)	0.006	(0.071)
Observations	141		94	
Pseudo-Log likelihood	−87.357		−37.659	

Notes: Single, double, and triple asterisks (*, **, and ***) denote significance level of 0.10, 0.05, and 0.01, respectively.

**Table 5:** Robustness check by excluding the outlier of preferences.

Variable	Marginal Effect	(Std. Err.)
Risk aversion (*σ*)	−0.709 ***	(0.042)
Loss aversion (*λ*)	0.062 ***	(0.013)
Probability weighting (*α*)	0.354 ***	(0.055)
Form	0.096	(0.069)
Pricing	0.111 **	(0.047)
Duration	0.032 **	(0.016)
Bonus	0.166 ***	(0.046)
Age	0.006 **	(0.003)
Education	0.005	(0.007)
Labor	−0.003	(0.016)
Asset	0.004	(0.008)
Scale	−0.001	(0.005)
Experience	−0.004 *	(0.002)
Fluctuation	0.097	(0.509)
Distance	−0.001	(0.001)
Region	−0.097 **	(0.040)
Observations	218	
Pseudo-Log likelihood	−49.075	

*Notes*: Single, double, and triple asterisks (*, **, and ***) denote significance level of 0.10, 0.05, and 0.01, respectively.

**Table 6:** Robustness check by using the survey questions of risk attitude.

Variable	Marginal Effect	(Std. Err.)
Risk attitude	−0.245 **	(0.017)
Form	0.130	(0.080)
Pricing	0.086 *	(0.049)
Duration	0.030 *	(0.018)
Bonus	0.179 ***	(0.046)
Age	0.006 **	(0.003)
Education	−0.0002	(0.007)
Labor	−0.036	(0.023)
Asset	0.006	(0.011)
Scale	0.001	(0.006)
Experience	−0.003	(0.003)
Fluctuation	−0.262	(0.608)
Distance	−0.004 **	(0.002)
Region	0.036	(0.002)
Observations	235	
Pseudo-Log likelihood	−78.585	

Notes: Single, double, and triple asterisks (*, **, and ***) denote significance level of 0.10, 0.05, and 0.01, respectively.
